# Effects of Osteoporosis on Bone Morphometry and Material Properties of Individual Human Trabeculae in the Femoral Head

**DOI:** 10.1002/jbm4.10503

**Published:** 2021-05-04

**Authors:** Martin Frank, Andreas G Reisinger, Dieter H Pahr, Philipp J Thurner

**Affiliations:** ^1^ Institute of Lightweight Design and Structural Biomechanics TU Wien Gumpendorfer Straße 7 Vienna 1060 Austria; ^2^ Department of Anatomy and Biomechanics, Division Biomechanics Karl Landsteiner University of Health Sciences Dr. Karl‐Dorrek‐Straße 30 Krems 3500 Austria

**Keywords:** AGING, BIOMECHANICS, BONE HISTOMORPHOMETRY, FRACTURE RISK ASSESSMENT, OSTEOPOROSIS

## Abstract

Osteoporosis is the most common bone disease and is conventionally classified as a decrease of total bone mass. Current diagnosis of osteoporosis is based on clinical risk factors and dual energy X‐ray absorptiometry (DEXA) scans, but changes in bone quantity (bone mass) and quality (trabecular structure, material properties, and tissue composition) are not distinguished. Yet, osteoporosis is known to cause a deterioration of the trabecular network, which might be related to changes at the tissue scale—the material properties. The goal of the current study was to use a previously established test method to perform a thorough characterization of the material properties of individual human trabeculae from femoral heads in cyclic tensile tests in a close to physiologic, wet environment. A previously developed rheological model was used to extract elastic, viscous, and plastic aspects of material behavior. Bone morphometry and tissue mineralization were determined with a density calibrated micro‐computed tomography (μCT) set‐up. Osteoporotic trabeculae neither showed a significantly changed material or mechanical behavior nor changes in tissue mineralization, compared with age‐matched healthy controls. However, donors with osteopenia indicated significantly reduced apparent yield strain and elastic work with respect to osteoporosis, suggesting possible initial differences at disease onset. Bone morphometry indicated a lower bone volume to total volume for osteoporotic donors, caused by a smaller trabecular number and a larger trabecular separation. A correlation of age with tissue properties and bone morphometry revealed a similar behavior as in osteoporotic bone. In the range studied, age does affect morphometry but not material properties, except for moderately increased tissue strength in healthy donors and moderately increased hardening exponent in osteoporotic donors. Taken together, the distinct changes of trabecular bone quality in the femoral head caused by osteoporosis and aging could not be linked to suspected relevant changes in material properties or tissue mineralization. © 2021 The Authors. *JBMR Plus* published by Wiley Periodicals LLC on behalf of American Society for Bone and Mineral Research.

## Graphical abstract

Individual trabeculae of human femoral heads were tested cyclically in tensile mode. Tensile modulus was determined in each loading and unloading cycle and significantly larger in the unloading phase. Determination of material properties with a rheological model revealed no significant difference between control and osteoporotic trabeculae. In contrast, bone morphometry was significantly deteriorated in osteoporosis and with increased ageing.

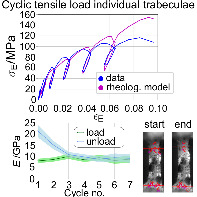

## Introduction

Osteoporosis is the most common bone disease and generally results in a decrease of total bone mass. According to WHO, osteoporosis is clinically defined as a decrease in areal bone mineral density (aBMD) by more than 2.5 SDs compared with young healthy controls.^(^ ^1^, ^2^ ^)^ aBMD measurement is done via DEXA at the femoral neck and the lumbar spine.^(^ ^3^ ^)^ Although patients are screened with this approach to determine need for treatment, only 60% of people that will suffer an osteoporotic fracture are correctly diagnosed.^(^ ^1^ ^)^ Accordingly, additional factors rather than aBMD alone are currently incorporated in diagnoses of osteoporosis, such as age, BMI, fracture history, cortisol treatment, epidemiologic information and other factors to calculate a 10‐year risk probability, the fracture risk assessment tool (FRAX) score.^(^ ^4^ ^)^ This score enables a more reliable risk prediction, but the underlying causes of increased fracture risk in osteoporosis still remain somewhat elusive and inaccessible for clinical diagnosis. Specifically, aBMD only reflects a combination of changes in bone mass and global mineralization, without accounting for bone quality. Bone quality, comprising all aspects aside of bone quantity, includes bone morphometry and tissue material properties, such as mechanical properties, material composition, and microdamage.^(^ ^5^ ^)^

Although there is common agreement that osteoporosis causes a change of bone morphometry,^(^ ^6^, ^7^, ^8^, ^9^ ^)^ conflicting results exist regarding changes in the tissue material properties.^(^ ^9^, ^10^ ^)^ Some studies determined a significant difference of material and mechanical properties in osteoporosis (lower Young's modulus,^(^ ^8^ ^)^ larger Young's modulus,^(^ ^11^, ^12^ ^)^ lower ultimate strain and postyield work^(^ ^13^ ^)^), whereas no difference was observed in ovariectomized animal models.^(^ ^14^, ^15^ ^)^ Similarly, opposing effects of osteoporosis on tissue mineral density (TMD) were reported, as being smaller,^(^ ^16^, ^17^, ^18^ ^)^ larger,^8^, ^11^, ^12^ ^)^ or unaffected,^(^ ^19^ ^)^ but accompanied by a larger heterogeneity of tissue mineralization.^(^ ^8^, ^19^ ^)^

Possible discrepancies are different species, anatomical sites, age, small donor and/or sample number, definition and/or severity of osteoporosis, different test methods and sample preparation, and focusing on elastic material behavior only. The current study aimed to address several of these issues by performing a thorough material characterization of the trabecular mechanical tissue properties, with a previously developed rheological model,^(^ ^20^ ^)^ in combination with bone morphometry. In that way the following two hypotheses could be investigated: First, the known morphological changes in osteoporosis are caused by changes in the mechanical tissue properties and tissue mineralization. Second, increasing age is additionally correlated with a deterioration of the trabecular network, associated with a decrease of the mechanical tissue properties.

## Materials and Methods

### Study design

The main goal of this descriptive study was to determine if there is a significant difference in the mechanical tissue properties between osteoporotic and control trabeculae. As such, six individual trabeculae obtained from a donor with a low trauma (osteoporotic) fracture (female, 77 years old) and six from a control cadaveric donor (male, 64 years old) were tested and evaluated in a pilot study in the same way as described in the following sections. Based on the obtained results, sample size (per group) was estimated with a power analysis at a significance level (α) of 95% (type I error: 0.05) and a power (β) of 80% (type II error: 0.20), according to Kadam and Bhalerao^(^ ^21^ ^)^ as: $n=\frac{{\left(Z_{\alpha /2}+Z_{1-\beta }\right)}^{2}2\sigma^{2}}{{\left(\mu_{1}-\mu_{2}\right)}^{2}}$ (whereby *Z*_*α*/2_: standard normal Z value, 1.96 for α = 0.05; *Z*_1 − *β*_: standard normal Z value, 0.84 for β = 80%; σ: pooled SD; μ_1_ – μ_2_: difference of means). Considering a dropout rate of 10% the required total number of samples (for both groups: $N=\frac{2n}{1-0.1}$) was 31 for apparent stiffness and 138 for apparent postyield work. Because a large biological and interdonor variation of the mechanical tissue properties is known from previous studies,^(^ ^22^, ^23^, ^24^ ^)^ we aimed to test 200 individual trabeculae in total, obtained from 20 donors (10 per group). Hereby, an equal number of male and female donors was selected per group (see Supplementary Information Table S1).

### Human bone samples and clinical data

Human femoral heads, together with clinical data (age, sex, BMI, aBMD, *T* score, and FRAX score) were obtained from a previous study^(^ ^25^ ^)^ and collected from two groups: osteoporotic fracture group (10 samples) and cadaveric control group (10 samples). Osteoporotic samples were obtained from patients undergoing hip arthroplasty at University Hospital Southampton NHS Foundation Trust (UHS) after a low trauma intracapsular fracture of the femoral neck. Sequentially, those patients suffered an actual osteoporotic fracture (which would not have occurred in healthy patients), which ensured bad bone quality. Cadaveric control samples were provided by Innoved Institute LLC (Besenville, IL). These donors had no known history of fracture or bone disease. In an additional second classification, grouping was based on *T* score, to avoid overlooking osteoporotic but nonfractured donors. *T* score was measured at the proximal femoral neck in vivo for fracture patients and with a modified approach for explanted cadaveric control specimens as described previously.^(^ ^25^ ^)^ Here, six donors were classified as osteoporotic (*T* < −2.5), six with osteopenia (−2.5 ≤ *T* ≤ −1.0), and eight as healthy controls (*T* > −1.0).

Full institutional review board and ethics approvals were obtained for the study (LREC 194/99/1; 210/01; 12/SC/0325) from the Southampton and South West Hampshire Research Ethics Committee.

### Sample dissection and individual trabeculae preparation

**Fig. 1:**
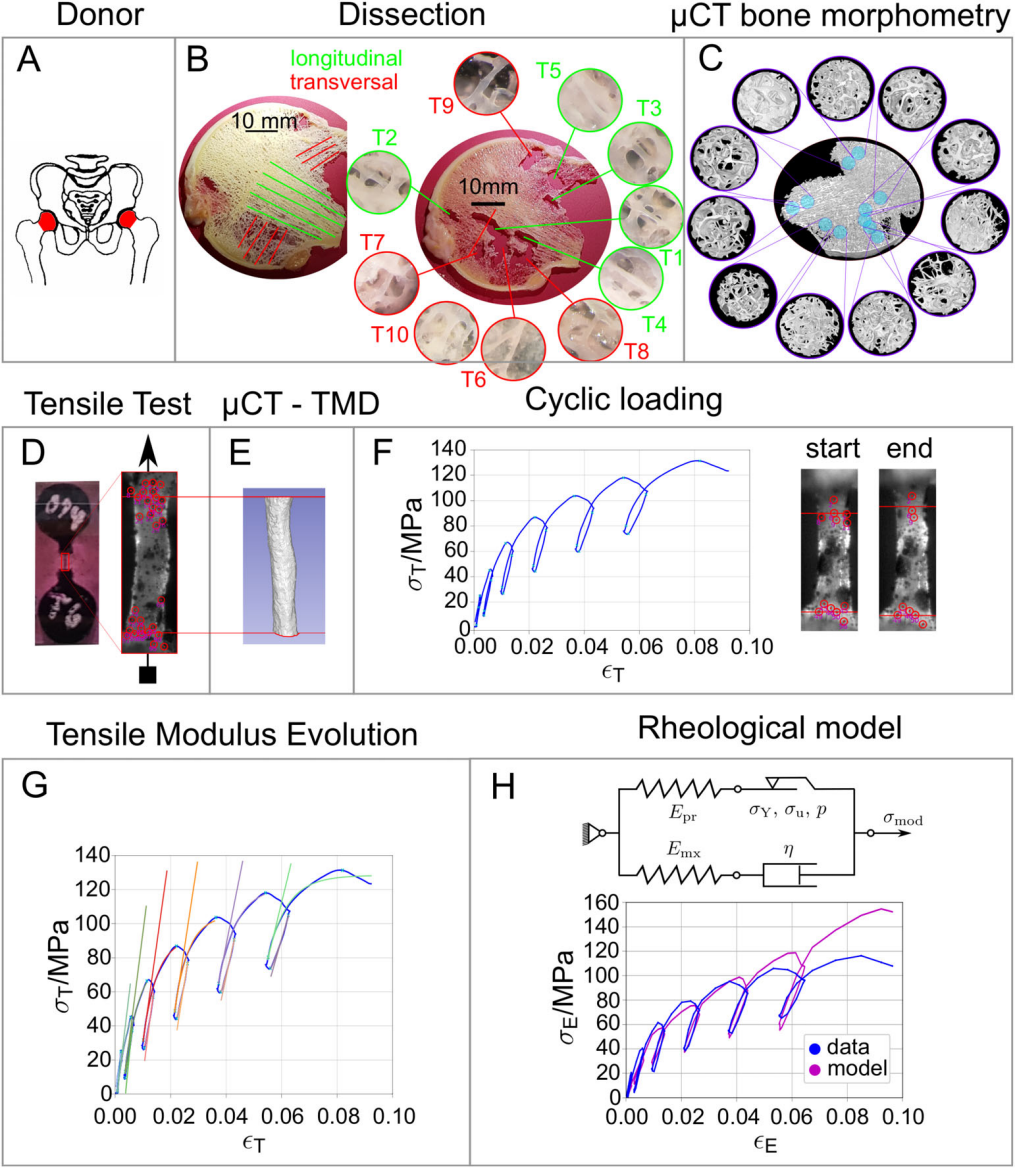
Operation chart: (*A*) Sampling locations from donors, femoral head. (*B*) Dissection of individual trabeculae in longitudinal (green) and transversal (red) direction. (*C*) μCT‐derived bone morphometry on the same locations, where individual trabeculae were dissected. (*D*) Tensile test sample (left, embedded in circular epoxy ends) and optical strain tracking (right, black speckle pattern). (*E*) μCT scanning of individual trabeculae to obtain geometry and tissue mineral density (TMD), with respect to the optically tracked region in the experiment. (*F*) True stress–strain curve obtained from cyclic loading. Insets show the trabecula at the start and end of the experiment. (*G*) Tensile modulus determined with exponential fits in each cycle. (*H*) Rheological model with determined engineering stress–strain curve.

Femoral heads were stored frozen at −80°C before usage. The samples were dissected in the frontal plane, using a band saw (Exakt), to obtain a 2‐mm‐thick slice (see Fig. 1 *A*). Individual trabeculae were extracted with a handheld miller (Dremel 400; Dremel Europe) under a stereo microscope (SZX10; Olympus Corp) as described in detail previously.^(^ ^26^, ^27^ ^)^ As the femoral head shows a typical arrangement of trabecular orientation, half of individual trabeculae were selected from the compressive, longitudinal trajectories (see Fig. 1*B*, green) and half from the arcuate, transversal trabecular system (red). Determination of dissection place specific bone morphometry was done ahead of actual dissection (see section “μCT: bone morphometry, trabecular geometry, and TMD” and Fig. 1*C*). Dissected individual trabeculae were placed in custom‐made silicone chambers and aligned properly using a light microscope (Zeiss Axio Imager; Carl Zeiss AG) for two orthogonal longitudinal planes. Sequentially, trabeculae were embedded with epoxy glue (UHU Endfest 300; UHU) to get tensile test specimens (see Fig. 1*D*). These specimens were sprayed with a spray paint (RAL9005; Dupli‐Color) to apply a speckle pattern for optical strain measurement.

### μCT: bone morphometry, trabecular geometry, and TMD

Ahead of bone dissection, the femoral heads were imaged using μCT with a μCT‐100 (Scanco Medical AG) at 70 kVp, 114 μA, integration time 200 ms, average data 3, 1500 projections, nominal resolution of 16 μm, and aluminum filter 0.5 mm. Image processing and determination of bone morphometry was done using medtool (version 4.3; Dr. Pahr Ingenieurs e.U.). The obtained images were segmented using a Gaussian filter (σ = 1, weight = 1) and a single‐level threshold of 490 mg/cm^3^ HA, ensuring that the “border layer” between Hank's balanced salt solution (HBSS) and bone is not included in the masked region. Sequentially, spheres with a diameter of 5 mm were cropped at the exact positions, where individual trabeculae were dissected (see Fig. 1*C*). This procedure enables a direct comparison of the obtained tissue mechanical data with the local bone morphometry. Bone volume to total volume (BV/TV), bone surface (BS), degree of anisotropy (DA), trabecular thickness (Tb.Th), trabecular number (Tb.N), and trabecular separation (Tb.Sp) were determined according to Bouxsein and colleagues^(^ ^28^ ^)^ and Dempster and colleagues.^(^ ^29^ ^)^

The same density‐calibrated μCT‐device mentioned above was used to obtain trabecular geometry and TMD of individual trabeculae (scanned in HBSS) with the same settings described above, but at a resolution of 3.3 μm. Calibration was done using five 6‐mm‐diameter hydroxyapatite cylinders of known density (0, 100, 200, 400, 800, all in mg/cm^3^ HA; whereby the 800 mg/cm^3^ HA phantom is measured weekly as control, using a standard protocol as provided by the manufacturer to ensure actual validity of the calibration). TMD was determined in whole individual trabeculae and in the fracture zone of the trabecular struts. In short, the fracture zone was classified as the whitened region (which is related to the region with microdamage accumulation^(^ ^30^ ^)^) in the last image of the video footage (point of fracture). Because of the speckle pattern, the borders of the whitened region in the image at fracture could be transferred to the corresponding borders in the image taken at the test's start. As the μCT images were taken ahead of sample testing, they could be registered manually onto the start video images in 3Dslicer (version 4.8.1; the Slicer Community) and cropped to the borders of the area that showed whitening during the test. Hereby, the mean normalized histograms (number of voxels divided by the total number of nonzero voxels) were determined on μCT images, which were masked with corresponding segmented images in medtool (version 4.3; Dr. Pahr Ingenieurs e.U.) to only consider voxels inside the trabeculae. Further, mean ± SD of TMD and heat plots of TMD were determined with Python scripts. Additionally, mean intensity profiles across the cross‐sectional mass centroid axes at the center of the fracture zone were computed. Here, length position was normalized by trabecular thickness. The generated 3D images were oriented according to the recorded 2D images from the experiment as mentioned previously.^(^ ^27^ ^)^ In short, the 3D μCT images were rotated in 3Dslicer (version 4.8.1; the Slicer Community) and cropped to the borders used for optical strain measurement (see Fig. 1*D,E*). The obtained volume was divided by its length to calculate a representative mean area for stress determination as described previously.^(^ ^26^ ^)^

### Mechanical tensile testing: stress and strain determination

Individual trabeculae were tested in tension in a water bath filled with HBSS to mimic a wet, physiologic environment.^(^ ^26^, ^27^ ^)^ A servo‐electric load‐frame (SELmini‐001; Thelkin AG) equipped with a 10‐N load cell (HBM‐S2M; HBM) was used. Long cylindrical samples (aspect ratio >3) were tested in pure tension to enable a proper material characterization at a defined, homogeneous stress state as reviewed in literature.^(^ ^31^ ^)^ Strain was determined optically, using a video camera (UI‐3250CP‐M‐GL, IDS GmbH), operated at 10 Hz, equipped with a KITO‐D zoom objective (mounted on a KITO‐ADP‐0.5 adapter, Kitotec GmbH). Video recording was done with μEye Cockpit (4.31, IDS Imaging Development Systems). Average gauge length was 687 ± 166 μm. Embedded tensile samples with illustration of optical strain tracking are displayed in Fig. 1*D*.

**Fig. 2:**
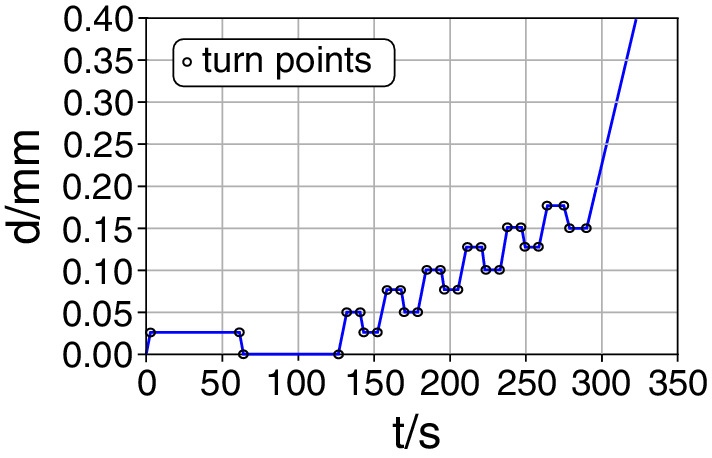
Loading profile was controlled by machine displacement (d) at a constant displacement rate of 0.01 mm/s for all cycles. In the first cycle, d is held constant for 60 s to ensure relaxation. Then, d is set to zero in the first unloading cycle and again held constant. Sequentially, d is increased by 0.025 mm in the next loading cycle, held constant for 10 s and decreased to d of the previous cycle in the unloading phase. This procedure is continued until fracture.

A cyclic‐loading regime (displacement driven) was selected to gain more information about the elasto‐visco‐plastic material behavior (see Fig. 2). In principle, the displacement was increased steadily with increasing cycle number and unloaded to the previous cycle displacement. Between the loading and unloading phases, the position was held constant for 10 s. This procedure continued until failure. Only the first two holding periods, after the first loading and unloading, lasted for 60 s, to ensure complete relaxation.

True stress was determined based on the mean cross‐sectional area of each trabeculae; logarithmic strain was determined optically using a point‐tracking algorithm and digital‐image correlation.^(^ ^27^ ^)^ In short, image series were cropped to the trabecular region and corrected for rigid body movement using ImageJ software (1.45 s; NIH; https://imagej.nih.gov/ij/). Next, a custom‐mode Python script (version 2.7) based on a point‐tracking algorithm^(^ ^32^, ^33^ ^)^ was used to determine displacement of points at the top and bottom region of the trabeculae. Strain was calculated between those points and averaged to get a mean vertical strain for each frame. A typical stress–strain curve is illustrated in Fig. 1*F*.

### Data evaluation: cyclic loading

Obtained stress–strain curves were segmented into individual cycles (containing loading, holding, and unloading phases). The turn points between loading and holding phases are marked as “o” in Fig. 2 and as “x” in Fig. 1*G*. A custom‐made Python script was used to obtain exponential fits *f*(*x*) for each loading ($f\left(x\right)=a∙\left(1-e^{\frac{x}{b}}\right)+c$) and unloading phase ($f\left(x\right)=\frac{a}{b}∙\left(e^{\mathrm{bx}}-1\right)$) in every cycle with the *curvefit* function (a nonlinear least squares approach) obtained from SciPy (version 0.18.0, the Scipy community). Furthermore, the first derivation was calculated analytically to compute the tangent of the obtained exponential fit (see straight colored lines in Fig. 1*G*) as the tensile modulus of each corresponding cycle.

### Data evaluation: the rheological model

For the extraction of further individual trabecular material properties from the experimental stress–strain data, a previously published inverse rheological modeling approach was used.^(^ ^20^ ^)^ This method is based on a two‐layer elasto‐visco‐plastic rheological model that is capable of reproducing the specimens' stress–strain response. It consists of an elasto‐plastic layer (Prandtl layer) and a visco‐elastic layer (Maxwell layer; see Fig. 1*H*). The Prandtl layer itself is built from an elastic spring with elastic modulus *E*_pr_ in series with a plastic slider that starts deforming upon reaching the yield stress (*σ*_y_). The yield stress is hardening exponentially—characterized by an exponent *p*—until plateauing at the ultimate stress (*σ*_u_). The Maxwell layer consists of an elastic spring with elastic modulus (*E*_mx_) in series with a viscous damper with a coefficient of viscosity (*η*).

The elastic moduli *E*_pr_ and *E*_mx_ can be interpreted as follows: For quasistatic deformation, the stress contribution of the Maxwell layer approaches zero and the model's stiffness is solely driven by the elastic spring in the Prandtl layer. Hence, *E*_pr_ can be referred to as the quasistatic or long‐term elastic modulus of the material (*E*_∞_). On the contrary, the apparent model stiffness reaches *E*_pr_ + *E*_mx_ for very high instantaneous deformations and is therefore referred to as the instantaneous elastic modulus (E_0_). So, *E*_pr_ and *E*_pr_ + *E*_mx_ represent the two bounds in‐between where any apparent stiffness of the material must reside when subjected to a finite strain rate.

The presented rheological arrangement also allows for a direct calculation of the loss tangent tan*δ* at an arbitrary excitation frequency. In this study, the loss tangent is evaluated at 1 Hz because it corresponds to the approximate frequency of human gait, besides allowing for a convenient comparison with other studies that report viscous properties of bone tissue also at that frequency.

For each tested trabecula, the set of material parameters [*E*_pr_, *σ*_y,_ *p*, *σ*_u_, *E*_mx_, *η*] is obtained in an inverse approach. The material parameters are tweaked using a downhill‐simplex algorithm with the objective function of minimizing the root mean square error (RMSE) between the experimental and model stress–strain response.

### Data evaluation: curve fitting

The apparent mechanical tissue properties ($\hat{E},{\hat{\varepsilon }}_{y},{\hat{\varepsilon }}_{u},{\hat{W}}_{\mathrm{el}},{\hat{W}}_{\mathrm{py}}$) were determined according to Frank and colleagues^(26,27)^ on the stress–strain envelope curve. In brief, apparent stiffness ($\hat{E}$) is determined with a linear regression on the envelope curve and yield strain (${\hat{\varepsilon }}_{y}$) is the end point of the maximum *R* ^2^ value of that regression. Elastic work (${\hat{W}}_{\mathrm{el}}$) is the area under the envelope curve until the yield point and postyield work (${\hat{W}}_{\mathrm{py}}$) is the area from yield until failure. For better discrimination, properties determined with the rheological model are referred as material properties and those determined with curve fitting as mechanical properties.

### Statistical analysis

Statistical data analysis was done in SPSS (version 26; IBM). First, data distribution was investigated with histograms, boxplots, and Q‐Q plots before selecting appropriate statistical tests as suggested by Lix and colleagues.^(^ ^34^ ^)^ Normality of data was further analyzed using a Kolmogorov–Smirnov test. Age, BMI, *R*, σ_y_, σ_u_, aBMD, trabecular length, BS, and Tb.N were normally distributed, whereas all other variables showed a nonnormal distribution. Thus, a Mann‐Whitney *U* nonparametric test was used for comparison of means for the fracture‐based classification (two groups). Similarly, a Kruskal‐Wallis nonparametric test was used for comparison of means for the *T* score–based classification (three groups) and for the analysis of the tensile modulus in each loading and unloading cycle (further referred as tensile modulus evolution (four groups: osteoporotic loading and unloading, control loading and unloading). In addition, a general linear model was used to determine if age is a contributing covariate in determining the mechanical or material properties, with fracture grouping or *T* score grouping as independent variables. Correlation was determined using the Spearman's rank correlation coefficient. Significance was accepted as *p* < 0.05 and a Bonferroni correction was applied for multiple testing.

**Fig. 3:**
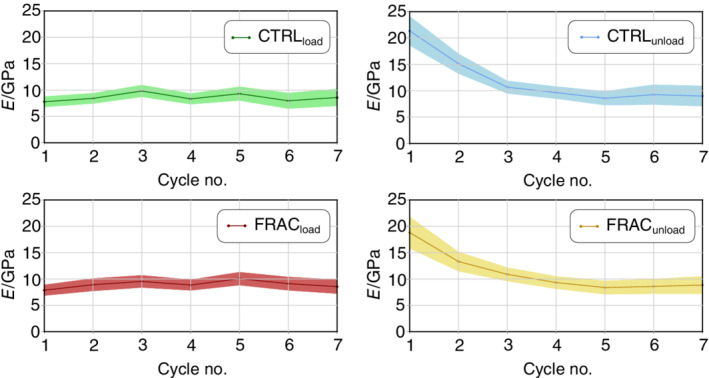
Tensile modulus for all cycles is shown as mean value with 95% CI, as shown in Fig. 1*G*. The loading modulus does not change significantly in any cycle neither in the osteoporotic fracture (FRAC) nor in the control group (CTRL). In contrast, there is a significant decrease of the unloading modulus from cycle 1 to 3 (*p* < 0.001) and 2 to 3 (*p* = 0.05) in the control group. Similarly, there is a significant decrease in the unloading modulus from cycle 1 to 3 (*p* = 0.011, and all subsequent cycles) in the osteoporotic group.

**Fig. 4:**
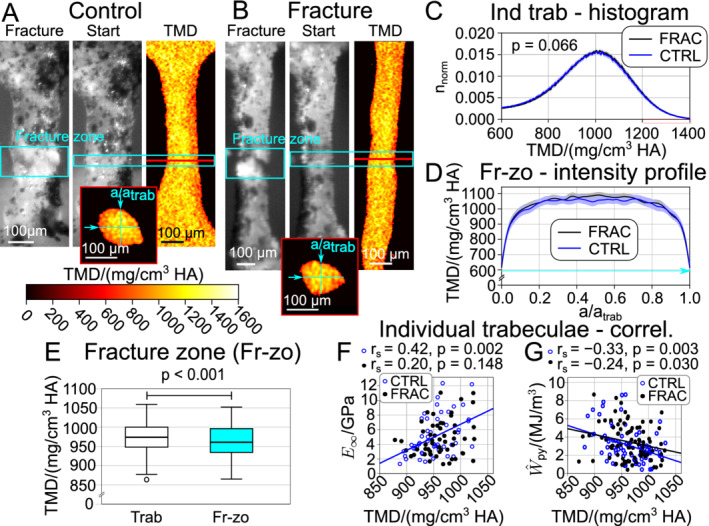
Tissue mineral density (TMD) distribution of individual trabeculae for osteoporotic fracture (FRAC)‐based classification (control [CTRL] blue, FRAC black). (*A*,*B*) Selected trabeculae, with optical tensile test recording at point of failure (left) and corresponding longitudinal TMD heat plot (determined from μCT images, taken ahead of mechanical testing), at central plane (right). The fracture zone is highlighted in cyan. Small insets at the bottom show the cross‐sectional TMD heat plot in the center of the fracture zone, as indicated in figures (*A*,*B*). (*C*) Normalized histogram of TMD distribution of all samples of FRAC and CTRL (mean solid, 95% CI shaded) of whole individual trabeculae (each value corresponds to one voxel obtained with μCT). (*D*) Mean intensity profile of all FRAC and CTRL samples (mean solid, 95% CI shaded) across the mass centroid axis (normalized), as illustrated in insets in subfigures (*A*,*B*). (*E*) Boxplot of all pooled samples in the fracture zone (cyan) and nonfractureds areas of corresponding whole individual trabeculae (each value is the mean of each whole trabeculae and fracture zone, p value determined with Wilcoxon signed rank test for pairwise samples). (*F*) Correlation plot of mean TMD with long‐term stiffness (E_∞_). (*G*) Correlation plot of mean TMD with apparent postyield work (W_py_). Abbreviations: Ind trab: individual trabeculae, Fr‐zo: fracture zone, correl: correlation.

As determination of material properties using the rheological model resulted in some unrealistic numbers, an interquartile range (IQR) test was performed to detect outliers, according to Reisinger and colleagues.^(^ ^20^ ^)^ First, this approach was applied on the pooled RMSE of the calculated stress signal to remove bad fittings. Next, only curves that showed at least three cycles before failure were used for further analysis. Then, all material parameters underwent an IQR test separately for each variable to remove unrealistic values. The variable‐based IQR test was also used for detection and removal of outliers in bone morphometry data and cyclic‐loading tensile moduli.

Comparison between inter‐ and intradonor variability of data was performed both with a Kruskal‐Wallis test and a one‐way ANOVA because data showed similar distributions, but deviated from a normal distribution. This procedure was performed to ensure that there was no influence from the assumed data distributions on the statistical outcome. It was hypothesized that interdonor variability (variability between individual donors) of given parameters is larger than intradonor variability (variability within individual donors).

## Results

In total, 179 individual trabeculae were successfully tested in cyclic tensile mode (89.5%, control: 90, fracture: 89; *T* > −1.0: 70, −1.0 > *T* > −2.5: 54, *T* < −2.5: 55). Clinical baseline characteristics are given in Table 1 (see Supplementary Information Fig. S1 for corresponding boxplots). Both grouping classifications indicated no significant difference in age and BMI. Patients that suffered from a low‐trauma fracture or with *T* < −2.5 had a significantly lower aBMD, *T* score, and FRAX score. No significant difference of the clinical, material, mechanical properties, or the bone morphometry was detectable between male and female donors (except a higher FRAX score and apparent yield strain in females; data not shown).

**Table 1:** Low‐Trauma (Fracture) and *T* Score Classifications for Clinical and Osteoporosis Factors. FRAX: fracture risk assessment tool score

Parameter	Fracture			*T* score			
	CTRL	FRAC	*p*	*T* > −1.0	−1.0 > *T* > ‐2.5	*T* < −2.5	K.W.
Age, y	69.5 ± 9.2	74.6 ± 11.0	0.307	68.1 ± 10.2	80.0 ± 7.5	71.3 ± 9.6	0.128
BMI, kg/m^2^	30.1 ± 9.2	26.1 ± 5.2	0.288	30.7 ± 9.5	29.4 ± 3.8	22.6 ± 3.2	0.068
*T* score	1.12 ± 2.94	−2.41 ± 0.83	**0.002**	1.70 ± 2.60	−2.14 ± 0.21	−2.92 ± 0.31^a^	**0.000**
FRAX, %	3.1 ± 3.4	13.9 ± 11.3	**0.031**	2.9 ± 3.6	13.2 ± 12.6	13.0 ± 10.8	**0.033**

*Note*. Mean values are indicated ± SD. Significant *p* values (*p* < 0.05) are marked in boldface. For *T* score–based classification, *p* values of the Kruskal‐Wallis test (K.W.) are noted and significant differences to *T* > −1.0 are marked with ^a^ in the corresponding column.

Abbreviations: CTRL, control; FRAC, osteoporotic fracture.

### Mechanical and material properties of trabecular bone tissue

Cyclic tensile modulus determination (see Fig. 1*G*) is only shown for the low‐trauma fracture classification, as the *T* score–based grouping showed the same trends. Evaluation was successful for 154 curves in cycle 1 (86.0%, control: 76, fracture: 78, *T* > −1.0: 59, −1.0 > *T* > −2.5: 45, *T* < −2.5: 50) and for 40 curves in cycle 7 (22.3%, control: 19, fracture: 21, *T* > −1.0: 13, −1.0 > *T* > −2.5: 21, *T* < −2.5: 6) because several samples had fractured after three cycles. The tensile modulus was significantly different between loading and unloading phase in the first two cycles (*p* < 0.001, for fracture and control groups, see Supplementary Information Table S2) and is illustrated in Fig. 3. In the control group, there was a significant decrease of the unloading modulus from cycles 1–3 (*p* < 0.001) and 2–3 (*p* = 0.05) and from cycles 1–3 in the fracture group (*p* = 0.011). After cycle 3, no significant changes in the tensile moduli were detectable. The loading modulus (control vs fracture) was 7.4 ± 4.2 versus 7.9 ± 4.7 GPa in the first cycle and did not change significantly in subsequent cycles. Interstingly, no significant difference between the control and fracture groups was detectable in any cycle.

The determination of material properties using the rheological model was successful in 107 curves (58.1%, control: 53, fracture: 54, *T* > −1.0: 43, −1.0 > *T* > −2.5: 32, *T* < −2.5: 32). As mentioned in the Statistical analysis subsection a strict selection regime was applied to only use reliable values. Ten curves (5.6%) were omitted because of the IQR test on the RMSE, 57 curves (31.8%) because of less than 4 successful test cycles and 14 values (7.8%; on average for all determined parameters) because of the variable‐specific IQR test. No significant difference in any mechanical or material property could be detected for both classifications (see Table 2 and Supplementary Information Figs. S2–S4 for corresponding boxplots), except a significantly larger apparent yield strain and elastic work between −1.0 > *T* > −2.5 and *T* < −2.5. Even the general linear model (with age as a covariate) did not change the statistical outcome, except for apparent yield stress in *T* score grouping. Further, selection of donor number as grouping variable indicated that long‐term modulus (*p* = 0.065) and ultimate stress (*p* = 0.049) have a larger variability between individual donors compared with intradonor variability.

**Table 2:** Low‐Trauma (Fracture) and *T* Score–Based Classifications for Study Parameters. *E*_∞_: long‐term modulus, *E*_mx_: Maxwell elastic modulus, *σ*_y_: yield stress, *p*: exponential hardening coefficient, *R*: hardening stress ($R=\sigma_{u}^{}‐\sigma_{y}^{}$), *σ*_u_: ultimate stress, *η*: viscosity, tan*δ*: loss tangent, Ê: apparent stiffness, ${\hat{\varepsilon }}_{y}$: apparent yield strain, ${\hat{\varepsilon }}_{u}$: apparent ultimate strain, ${\hat{W}}_{\mathrm{py}}$: apparent post‐yield work, ${\hat{W}}_{\mathrm{el}}$: apparent elastic work.

Parameter	CTRL	FRAC	*p*	*T* > −1.0	−1.0 > *T* > −2.5	*T* < −2.5	K.W.
*E*_∞_ , GPa	5.0 ± 2.7	4.9 ± 2.5	0.872	4.9 ± 2.7	5.5 ± 2.9	4.5 ± 2.1	0.438
*E*_*mx*_ , GPa	2.4 ± 1.3	2.6 ± 1.5	0.474	2.3 ± 1.3	2.8 ± 1.5	2.6 ± 1.5	0.351
*σ*_*y*_ , MPa	30.8 ± 18.2	31.9 ± 19.8	0.813	30.8 ± 19.3	35.5 ± 20.6	28.1 ± 16.3	0.359
*p*	62.7 ± 58.5	63.3 ± 66.2	0.991	59.5 ± 61.6	69.3 ± 63.9	61.0 ± 63.4	0.654
*R*, MPa	59.4 ± 30.0	61.0 ± 26.9	0.517	62.7 ± 35.5	59.4 ± 20.7	57.9 ± 26.2	0.997
*σ*_*u*_ , MPa	84.3 ± 29.4	93.8 ± 38.6	0.133	84.2 ± 34.1	93.4 ± 29.6	90.3 ± 39.7	0.343
*η*, GPas	4.8 ± 3.8	4.3 ± 3.2	0.665	5.4 ± 3.9	4.4 ± 3.6	3.7 ± 2.5	0.193
tan*δ*	0.017 ± 0.011	0.021 ± 0.013	0.087	0.019 ± 0.011	0.019 ± 0.012	0.020 ± 0.014	0.948
$\hat{E}$, GPa	8.5 ± 5.1	7.7 ± 4.4	0.408	7.5 ± 4.3	9.9 ± 5.7 ^b^	7.1 ± 4.0	**0.030**
${\hat{\varepsilon }}_{y}$, %	0.22 ± 0.16	0.27 ± 0.21	0.280	0.23 ± 0.17	0.19 ± 0.17^b^	0.31 ± 0.21	**0.002**
${\hat{\varepsilon }}_{u}$, %	5.0 ± 2.2	5.5 ± 2.4	0.159	5.1 ± 2.2	5.2 ± 2.6	5.4 ± 2.2	0.615
${\hat{W}}_{\mathrm{py}}$, MJ/m^3^	3.0 ± 1.9	3.4 ± 1.9	0.157	3.0 ± 1.8	3.3 ± 2.0	3.6 ± 2.0	0.215
${\hat{W}}_{\mathrm{el}}$, MJ/m^3^	0.018 ± 0.017	0.023 ± 0.022	0.274	0.019 ± 0.019	0.014 ± 0.019^b^	0.027 ± 0.022	**0.001**

*Note*. Mean values are indicated ± SD. K.W. denotes the *p* value obtained with the Kruskal‐Wallis test. Significant *p* values (*p* < 0.05) are highlighted in boldface.

Abbreviations: CTRL, control; FRAX, fracture.

^b^Illustrates a significant (*p* < 0.05) difference to *T* < −2.5.

Additionally, samples were grouped according to their orientation along the trajectories (longitudinal vs transversal; see Fig. 1*B*). No significant difference in any mechanical or material property could be determined, except larger apparent yield strain (${\hat{\varepsilon }}_{y}$) and apparent elastic work (${\hat{W}}_{\mathrm{el}}$) for transverse trabeculae (see Supplementary Information Table S3). This difference could be related to a significant correlation (*p* < 0.001) of ${\hat{\varepsilon }}_{y}$ and ${\hat{W}}_{\mathrm{el}}$ with average cross‐sectional area (A_mean_, r_s_ = −0.60 and −0.64 on pooled data). A_mean_ of transverse trabeculae was significantly smaller compared with longitudinal ones (0.016 ± 0.007 mm^2^ vs 0.022 ± 0.010 mm^2^; *p* < 0.001), and thus indicated that the smaller, transversal trabeculae yield at larger strains.

### Tissue mineral density

TMD distribution of whole individual trabeculae did not differ significantly in the fracture‐based classification (see Table 3 and Fig. 4 *A*‐*C*). However, in *T* score–based grouping TMD of whole trabeculae was significantly larger in the group −1.0 > *T* > −2.5, compared with *T* > −1.0 and *T* < −2.5 (see Table 3 and Supplementary Information Fig. S5). In contrast, in the fracture zone there was neither a difference in histograms of TMD between sample groups using both classifications (p = 0.172; see Table 3), nor in intensity profiles (see Fig. 4 *D*). Mean TMD was lower in the fracture zone in comparison with the nonfractured part of corresponding whole individual trabeculae (see Fig. 4 *E* and Table 3; *p* < 0.001 for pooled data). The average trabecular diameter (determined from the average cross‐sectional area, assuming a circular cross‐section) was not significantly different between the fracture zone (156 ± 41μm) and the nonfracture zone (149 ± 38 μm; *p* = 0.696). TMD showed a significant positive correlation with long‐term stiffness (E_∞_, r_s_ = 0.42, *p* = 0.002 for control samples and r_s_ = 0.30, *p* = 0.002 for pooled data; see Fig. 4*F*) and with all tensile moduli (cycles 2–7, loading and unloading). Ultimate strain (r_s_ = −0.33, *p* < 0.001; see Supplementary Information Fig. S6) and postyield work (r_s_ = −0.29, *p* < 0.001; see Fig. 4*G*) were significantly negatively correlated with TMD, based on pooled data.

### μCT‐derived bone morphometry

The fracture‐based classification (see Table 4) indicated a significant difference with smaller values of BS and Tb.N for the fracture group. In contrast, BV/TV, Tb.Sp, Tb.Th, and DA were not affected. Osteoporotic samples based on the *T* score (see Table 4) showed significantly lower values of BV/TV, BS, and Tb.N compared with *T* > −1.0. In contrast, Tb.Sp was significantly higher and DA and Tb.Th remained unaffected (see Supplementary Information Figs. S7 & S8 for corresponding boxplots and Supplementary Information Fig. S8 for representative bone slices). Bone morphometry did not differ significantly between longitudinal and transversal trabeculae, except DA (1.64 ± 0.22 vs 1.57 ± 0.25 vs, *p* = 0.046). Bone morphometry showed no significant correlation with mechanical properties but with material properties, namely for Tb.Sp versus R (r_s_ = 0.24, *p* = 0.034), Tb.Sp versus σ_u_ (r_s_ = 0.24, *p* = 0.031), and Tb.Th versus σ_y_ (r_s_ = 0.25, *p* = 0.011) on pooled data. Furthermore, bone morphometry parameters indicated a significantly larger interdonor variability, compared with intragroup variance (estimated with two‐side ANOVA and Kruskal‐Wallis test, *p* < 0.001).

**Table 3:** Low‐Trauma (fracture) and *T* Score–Based Classifications for Tissue Mineralization Density in the Fracture Zone and Whole Individual Trabeculae

Location	Fracture			*T* score			
	CTRL	FRAC	*p*	*T* > −1.0	−1.0 > *T* > −2.5	*T* <−2.5	K.W.
Fracture zone	951 ± 174	954 ± 173	0.172	953 ± 175	956 ± 168	950 ± 177	0.172
Individual trabeculae	958 ± 174	963 ± 173	0.066	960 ± 175	968 ± 167^a,b^	955 ± 176	**0.001**

*Note*. Mean values are indicated ± SD. K.W. denotes the *p* value obtained with the Kruskal‐Wallis test. Significant *p* values (*p* < 0.05) are highlighted in boldface.

Abbreviations: CTRL, control; FRAC, fracture.

^a^Illustrates a significant (*p* < 0.05) difference to *T* > −1.0.

^b^Illustrates a significant (*p* < 0.05) difference to *T* < −2.5.

### Age‐related changes

**Fig. 5:**
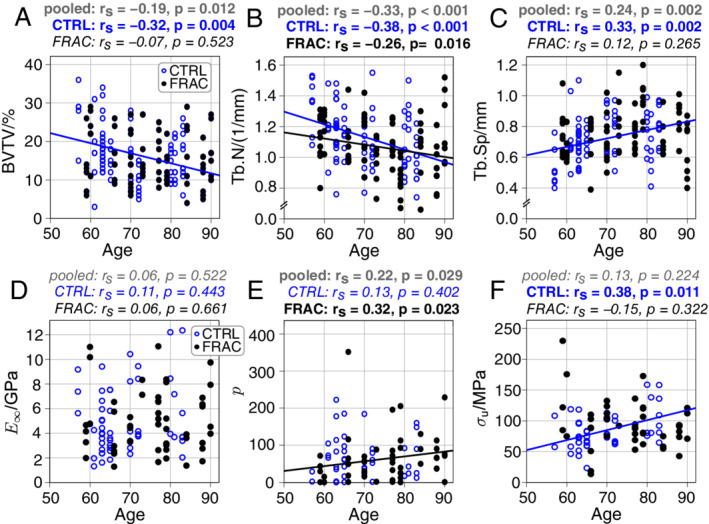
Correlation of μCT‐derived bone morphometry (top panels, *A*–*C*) and material tissue properties (bottom panels, *D*–*F*) with age. (*A*) Bone volume to total volume (BV/TV) decreased significantly with increasing age only for control trabeculae (CTRL). (*B*) Trabecular number (Tb.N) decreased significantly with increasing age. (*C*) Trabecular separation (Tb.Sp) increased significantly with increasing age only for CTRL. (*D*) Long‐term modulus (E_∞_) showed no significant correlation with age. (E) Hardening exponent (p) increased significantly with age only for osteoporotic fracture trabeculae (FRAC). (*F*) Ultimate stress (σ_u_) increased significantly with age only for CTRL. Spearman rank correlation coefficients are given on top of each panel, with actual *p* values and highlighted bold, if significant. Linear regression lines are provided for significant correlations.

Age‐related changes of mechanical and material properties and bone morphometry were determined both on pooled data (to check the influence of age together with osteoporosis) and on nonfractured samples (to determine the influence of aging independently from osteoporosis; see Fig. 5). For pooled data Tb.N (r_s_ = −0.33; *p* < 0.001), Tb.Sp (r_s_ = 0.24; *p* = 0.002), BV/TV (r_s_ = −0.19; *p* = 0.012), and BS (r_s_ = −0.32; *p* < 0.001) showed a significant dependency on age, whereas all other mechanical and material properties (except exponential hardening coefficient p, r_s_ = 0.22, *p* = 0.029) did not correlate with age. Here, the exponential hardening coefficient showed a moderate correlation (r_s_ = 0.32, *p* = 0.023) with age for fracture group, whereas control trabeculae were not correlated with age. In control trabeculae, age was significantly correlated with Tb.N, Tb.Sp, and BV/TV. Further, control trabeculae showed a moderate correlation of age with ultimate stress (r_s_ = 0.38; *p* = 0.011). Donor age was not correlated with TMD for pooled data and both grouping classifications.

## Discussion

This study found comprehensive mechanical and material properties (elastic, viscous, and post yield) of individual osteoporotic and healthy trabeculae, loaded cyclically, in tension close to a wet, physiologic environment. Inspired by work on micropillar compression on cortical bone,^(^ ^35^ ^)^ our study elucidates the significant difference between loading and unloading moduli for trabecular bone tissue. This difference can be explained with viscous effects and damage accumulation. Indeed, the selection of different viscosities and holding periods in the rheological model could reproduce differences in the loading and unloading moduli (data not shown). Further, it is speculated that damage initiation appears in the end phase of the second and third loading cycle, causing a drop of subsequent unloading stiffnesses. According to the three‐phase model of Fazzalari and colleagues,^(^ ^36^ ^)^ damage growth does not cause a decrease in stiffness, which might explain why loading stiffness (determined at the initial part of each loading cycle) stays almost constant. As only one‐to‐two damage sites are initiated per trabeculae^(^ ^37^ ^)^ crack/damage growth might be dominant, instead of crack/damage initiation, explaining the constant unloading stiffness in later cycles.

This makes clear an important fact: Human trabecular bone tissue is not linear elastic by nature, as already observed in nanoindentation experiments.^(^ ^38^, ^39^ ^)^ In fact, our results shine some light on the discrepancy in material properties derived from nanoindentation and tests of individual trabeculae; nanoindentation looks at the unloading part whereas mechanical tests of individual trabeculae generally consider the loading part. Additionally, nanoindentation is most often performed on dry samples, which further increases the discrepancy. This puts forth an important question: How do we deal with trabecular bone in computational models? Is a linear elastic approach sensible? This will largely depend on what is being modeled; however, it should be clear that such linear elastic approaches can barely depict true mechanical behavior and do not deliver insight on actual material properties.

A main goal of the presented study was to perform a reliable characterization of osteoporotic trabeculae, in comparison to a healthy age‐matched cohort. Trabeculae were taken from the longitudinal trajectories and from the transversal arcuate region, to discriminate potential differences in mechanical and material properties. However, no significant difference in mechanical or material properties was detected, except a higher apparent yield strain, apparent elastic work and a lower TMD (*p* = 0.02) in transversal trabeculae. Interestingly. Torres and colleagues mentioned that transversal oriented trabeculae serve as sacrificial elements and enhance fatigue life of cancellous bone^(^ ^40^ ^)^; this could be partly related to the increased elastic deformation of individual trabeculae, probably caused by decreased mineralization.

In total, 179 trabeculae from 20 donors were successfully tested to ensure a good representation of actual mechanical and material properties, as a large biological variation^(^ ^22^, ^23^, ^24^ ^)^ and technical difficulties in micromechanical testing^(^ ^41^ ^)^ are known. Indeed, we determined a coefficient of variation (COV) of 0.54 and 0.46 for long‐term and instantaneous elastic modulus, comparable to COV values from previous studies on tensile tests of individual trabeculae ranging from 0.15 to 0.74.^(^ ^13^, ^26^, ^27^, ^42^, ^43^ ^)^ Reliability of the determined material properties, obtained with the rheological model, has already been found previously for healthy human trabeculae.^(^ ^20^ ^)^

Taking this large variation into account, no significant differences in the material properties or mineralization were detected between osteoporotic trabeculae (in both classifications) compared with healthy control ones, even if age was considered as a covariate. However, apparent stiffness and TMD were significantly larger for donors with osteopenia (−1.0 < T < −2.5), whereas apparent yield strain and elastic work were significantly smaller compared with osteoporotic donors (T < −2.5). For pooled data, TMD was significantly negatively correlated with apparent yield strain (r_s_ = −0.36), elastic work (r_s_ = −0.33), and postyield work (r_s_ = −0.29, *p* < 0.001), and positively with tensile modulus, long‐term ealstic modulus, and Maxwell elastic modulus (r_s_ = 0.30 on average, *p* = 0.002). Apparently, increased TMD levels cause a stiffening of trabeculae, associated with a decrease of elastic work in patients with osteopenia. However, this effect is diminished in osteoporotic patients because mechanical properties might depend on the duration of estrogen deficiency, as shown in ovariectomized sheep.^(^ ^16^ ^)^ In the current study, average osteoporotic donor age was 74.6 years and prolonged estrogen deficiency might have diminished initial differences in elastic tissue properties of female donors. It has to be pointed out that trabeculae from donors with osteopenia had a significantly larger cross‐sectional area compared with osteoporotic and control samples; and only apparent (geometry‐dependent) mechanical properties (yield strain, elastic work, and Young's modulus) were affected. In contrast, none of the actual material properties (determined via the rheological model) were different. A possible explanation might be that the apparent properties show a larger dependency on the geometry than the rheological model. The geometry dependency might further explain the large variation of previously reported values in the literature and would favor the determination of material properties with models minimizing the influence of sample geometry.

In the literature, no consensus has been reached so far on the influence of age and disease on trabecular tissue properties.^(^ ^44^ ^)^ On the one hand, no change of material properties of trabecular tissue of humans experiencing an osteoporotic fracture,^(^ ^45^, ^46^ ^)^ of ovariectomized rats,^(^ ^14^, ^47^ ^)^ and of postmenopausal women^(^ ^48^ ^)^ was determined. On the other hand, decreased mechanical properties in osteoporotic donors^(^ ^8^ ^)^ and ovariectomized sheep,^(^ ^16^, ^49^ ^)^ as well as increased mechanical properties in ovariectomized rats^(^ ^11^, ^12^ ^)^ have been reported. Possible discrepancies might be related to differences between donors with osteopenia and osteoporosis, as observed in our study at least for the apparent linear region. Further discrepancies are comparisons with not age‐matched controls,^(^ ^8^ ^)^ reporting only apparent properties, testing on dried specimens, and different test procedures. The tensile test used in the present study enables a well‐defined homogeneous stress state in contrast to three‐point bending, which has been shown to give different results than tensile tests for obtained material properties.^(^ ^50^ ^)^ Furthermore, small donor numbers might cause artificial differences between control and osteoporotic mechanical and material properties, as variability between donors has already been reported as being much larger than inside a single donor,^(^ ^24^ ^)^ possibly caused by differences in TMD and the remodeling state.^(^ ^41^ ^)^ In the present study, interdonor variability was also significantly larger for ultimate stress (*p* = 0.049) and almost for long‐term modulus (*p* = 0.065).

In contrast to previous studies,^(^ ^8^, ^19^ ^)^ no increased heterogeneity of mineralization of osteoporotic trabeculae could be detected. Only smaller TMD values at the trabecular surface, compared to the center, have been observed (as reported previously in Brennan et al.^(^ ^19^ ^)^ and Mulder et al.^(^ ^51^ ^)^), without being different across groups. Hereby, the outermost layer might be influenced by the partial volume effect, but as TMD was lower in the outermost three voxels of the surface (see Fig. 4), this phenomenon cannot be solely described by the partial volume effect. Previously, TMD was found to be increased^(^ ^11^, ^12^ ^)^ or decreased^(^ ^16^, ^17^, ^18^, ^52^ ^)^ in osteoporosis, but different measurement techniques were used (μCT, quantitative backscattered electron imaging (qBEI), Fourier transform infrared microspectroscopy) and different anatomical sites investigated. Interestingly, mean TMD was significantly lower in the fracture zone in comparison with the nonfractured part of the corresponding whole individual trabeculae (*p* < 0.001 pairwise comparison for pooled data; see Fig. 4 *E*). In general, TMD was always lower in the fracture zone, irrespective of grouping (see Table  3). This finding agrees with Turunen and colleagues,^(^ ^53^ ^)^ where TMD was also significantly lower at crack locations in comparison with surrounding trabecular bone. However, Turunen and colleagues^(^ ^53^ ^)^ could relate the lower TMD to a significantly lower trabecular thickness in the fracture zone. Surprisingly, in the current study trabecular diameter was not different from the nonfracture region. It is assumed that trabecular thickness is a good estimator of crack initiation at the level of trabecular bone structures (which trabeculae are likely to fail), but not for the exact locations inside individual trabeculae (where it will fail exactly). Here, lower mineralized regions might exhibit larger strains because of the smaller local stiffness, and might thus be the initiation points of damage with elevated stress levels. Qualitative doublechecking of the video footage revealed that most trabeculae did indeed not fracture at the thinnest location. It has to be mentioned that the found correlations of TMD with mechanical properties, although being similar to previously reported values for tissue (Young's modulus^(^ ^27^, ^54^, ^55^, ^56^ ^)^ and postyield work^(^ ^50^ ^)^), are only moderate (~0.30). This highlights that other factors, such as porosity (lacunae, microcracks)^(^ ^8^ ^)^ and the collagen phase^(^ ^57^, ^58^, ^59^ ^)^ play a relevant additional role in the determination of mechanical and material properties, which were not assessed in the current study.

**Table 4:** Low‐Trauma (Fracture) and *T* Score–Based Classifications for Study Parameters. BS: bone surface, BV/TV: bone volume to total volume, Tb.N: trabecular number, Tb.Sp: trabecular separation, Tb.Th: trabecular thickness, DA: degree of anisotropy

Parameter	Fracture			*T* Score			
	CTRL	FRAC	*p*	*T* > −1	−1 > *T* > −2.5	*T* < −2.5	K.W.
BS, mm^2^	185.4 ± 51.2	164.7 ± 45.4	**0.010**	197.9 ± 49.4	166.2 ± 45.7^a^	155.1 ± 41.3^a^	**0.000**
BV/TV, %	16.8 ± 7.0	15.5 ± 6.7	0.186	18.4 ± 7.0	16.0 ± 6.4	13.5 ± 6.2^a^	**0.000**
Tb.N, 1/mm	1.13 ± 0.19	1.07 ± 0.18	**0.046**	1.17 ± 0.18	1.04 ± 0.19^a^	1.07 ± 0.18^a^	**0.000**
Tb.Sp, mm	0.72 ± 0.15	0.77 ± 0.16	0.128	0.69 ± 0.14	0.78 ± 0.16^a^	0.78 ± 0.15^a^	**0.001**
Tb.Th, mm	0.17 ± 0.03	0.17 ± 0.03	0.466	0.17 ± 0.03	0.17 ± 0.03	0.17 ± 0.04	0.311
DA	1.59 ± 0.24	1.62 ± 0.23	0.215	1.59 ± 0.25	1.65 ± 0.20	1.58 ± 0.25	0.138

*Note*. K.W. denotes the *p* value obtained with the Kruskal‐Wallis test. Significant *p* values (*p* < 0.05) are highlighted in boldface.

Abbreviations: CTRL, control; FRAC, fracture.

^a^Illustrates a significant (*p* < 0.05) difference to *T* > −1.0.

The absence of changes of mechanical and material properties and mineralization with aging is in accordance with previous findings about tissue Young's modulus,^(^ ^60^ ^)^ viscoelastic properties,^(^ ^61^ ^)^ and TMD.^(^ ^62^ ^)^ However, increased mineralization with increasing age has also been reported,^(^ ^16^, ^63^, ^64^ ^)^ possibly related to a slowed‐down bone turnover in the elderly.^(^ ^65^ ^)^ As only rod‐shaped trabeculae have been investigated in the present study, it might be that mineralization in plate‐shaped trabeculae is more affected by aging because mineralization is significantly different between these two trabecular shapes.^(^ ^66^ ^)^ Only tissue strength was increased with increasing age in the present study for control trabeculae. In contrast with previous studies,^(^ ^16^, ^63^, ^64^ ^)^ no increased mineralization in the elderly has been detected, suggesting that other factors are additionally responsible for tissue stiffening, at least in the age range studied here. Interestingly, exponential hardening coefficient was increased with increasing age (r_s_ = 0.32, *p* = 0.023) for osteoporotic fracture donors only. Hence, aging may affect material properties differently between healthy and osteoporotic donors. Although all other mechanical and material properties were independent of donor age, they might be dependent on tissue age,^(^ ^67^, ^68^ ^)^ which could be assessed in future studies, for example, via qBEI.

In contrast with most material properties, bone morphometry was significantly affected by osteoporosis and aging. Osteoporotic samples showed a significantly smaller BV/TV, BS, and Tb.N, accompanied with a significantly larger Tb.Sp, without affecting Tb.Th. Accordingly, a smaller BV/TV,^(^ ^6^, ^8^, ^69^ ^)^ Tb.N,^(^ ^6^, ^8^, ^69^, ^70^ ^)^ and a larger Tb.Sp^(^ ^6^, ^8^, ^69^, ^70^ ^)^ have been reported in the literature for osteoporotic trabecular bone. Tb.Th has been noted to be controversial; it has been reported to be unaffected^(^ ^6^, ^8^ ^)^ or larger.^(^ ^7^ ^)^ Interestingly, we could only find a few weak correlations (r_s_ ≤ 0.25) of bone morphology parameters with material properties (for pooled data: Tb.Sp vs R, Tb.Sp vs σ_u_, and Tb.Th vs σ_y_), suggesting that bone structure only depends at most weakly on material properties. No significant differences in bone morphometry parameters were observed between male and female donors. Both groups displayed large interdonor variability. For the female donors one can speculate that this may partly be caused by individual differences in the duration and intensity of metabolic changes. For example, prolonged estrogen deficiency has been reported to decrease BV/TV,^(^ ^71^ ^)^ Tb.N,^(^ ^72^ ^)^ Tb.Th,^(^ ^71^ ^)^ and increase Tb.Sp^(^ ^71^, ^72^ ^)^ in the ovariectomized rat model. Additionally, BV/TV, BS, Tb.N, and Tb.Sp indicated a significant dependency on age, as reported previously for BV/TV,^(^ ^69^, ^73^, ^74^ ^)^ Tb.Sp,^(^ ^69^, ^73^ ^)^ Tb.N,^(^ ^69^, ^73^, ^74^ ^)^ Tb.Th,^(^ ^75^ ^)^ and degree of anisotropy.^(^ ^75^ ^)^ BV/TV between control and osteoporotic samples (fracture grouping) was not significantly different, whereas the *T* score was. The *T* score was determined at the femoral neck; BV/TV was determined at the exact locations of trabecular dissections in the femoral head (see Fig. 1*C*), which might explain this difference. Furthermore, in the general linear model, with age as a covariate, BV/TV was also significantly lower in the fracture group. BV/TV was significantly correlated with age in the control group, but not for osteoporotic (fractured) samples. These data suggest that bone morphometry changes differently between normal aging and osteoporosis. This finding is in agreement with Boskey and colleagues who stated that osteoporosis is associated with aging, but is not a cause of aging.^(^ ^76^ ^)^

Micromechanical tests inherit several limitations caused by the small, irregular sample geometry and difficulties in sample handling as has been intensively reviewed^(^ ^31^ ^)^ and has been further mentioned in previous studies.^(^ ^8^, ^27^, ^50^, ^77^ ^)^ Thus, testing a large number of samples with an aspect ratio larger than three was performed to obtain a reasonable number of representative samples. Misalignment of samples causes shear stress^(^ ^41^ ^)^ and obtained values might be erroneous. Consequently, special care was taken to align samples using images obtained from two orthogonal planes. Furthermore, heterogeneity in stresses might evolve from irregularly shaped trabeculae. This was minimized by the selection of long cylindrical trabeculae. Previously, compressive loading of human femoral head plugs was found to result in compressive and tensile strains.^(^ ^53^ ^)^ Because the compressive material properties could be potentially differently affected by osteoporosis, this issue should be investigated in the future. Only rod‐shaped (likely old) trabeculae were tested. Plate‐shaped (likely younger samples) might be affected differently because of differences in the remodeling rate, similar to previously detected differences of TMD between longitudinal plate trabeculae and rod‐shaped transversal samples.^(^ ^66^ ^)^ Another limitation is the optical strain tracking, especially in the first cycle; the signal‐to‐noise ratio is lower at low strains. In addition, samples were tested in displacement control because of technical limitations and a more efficient test procedure, whereas strain or stress control would enable a more uniform loading protocol. As trabecular length was not constant, the number of cycles until fracture differed. Smaller samples fractured after three cycles because of larger strains. Thus, it is recommended to use a pseudostrain‐driven–loading profile (the set displacement should be dependent on individual trabecular length) for future studies. Addressing this issue would also increase the number of useable stress–strain curves for the rheological model (as samples would fracture at later cycles and as only samples that were tested for at least four cycles were included in this evaluation). A further limitation was that potential changes of the collagen phase were not investigated in the course of the present study. Previous studies have highlighted the importance of collagen in determining bone mechanical properties^(^ ^58^, ^59^, ^78^ ^)^; this should also be evaluated in future studies for individual trabeculae. The determination of TMD using μCT is known to have a relatively good correlation with ash density, but also to underestimate true TMD values and to show a geometry dependency.^(^ ^79^, ^80^ ^)^ μCT‐derived bone morphometry was determined at the exact points of trabecular dissection for correlation purposes with mechanical and material properties. Spheres were used to avoid misalignment of the ROI, whereas cubes are more common in bone morphometry. Still, comparable values for cubes of trabecular bone in the femoral head^(^ ^81^ ^)^ were obtained and slightly smaller values for BV/TV and Tb.N are possibly caused by the selection of regions with a low BV/TV because they contain a larger amount of cylindrical trabeculae.

## Conclusions

The observed weakening of the apparent mechanical behavior of trabecular bone in osteoporosis is likely largely caused by changes of the bone morphometry and to a lesser extent by weakening of the tissue itself, at least for the femoral head. Similarly, aging in our study also caused a deterioration of bone morphology without affecting material properties, except for an increase of tissue strength and exponential hardening coefficient. Because these two variables and BV/TV were differently affected between healthy and osteoporotic trabeculae, it is assumed that age‐related changes are different in osteoporosis and healthy cohorts. The substantial variation of obtained material and mechanical properties suggests that small differences between healthy and osteoporotic trabeculae could not be detected. Given the large variability of the detected material properties (inter‐ and intradonor variability), a general statement of their relevance for clinical cohort classification cannot be made. Nevertheless, the large variation could affect the mechanical competence in an individual patient regardless of a classification as healthy, osteopenic, or osteoporotic (e.g., per a BMD measurement). Sequentially, it might be favorable to use valid boundary values of material parameters (e.g., mean ± SD) for the estimation of total bone strength in computer simulations instead of average values. Detected differences in apparent yield strain and elastic work of donors with osteopenia might be related to differences at disease onset and the inherent geometrical dependency of apparent mechanical properties. The presented study is the first that has determined the actual material (not only apparent) properties of healthy and osteoporotic trabeculae and has highlighted that trabecular bone tissue is an elasto‐visco‐plastic material and cannot be described properly as being linear‐elastic. This finding is important for computer simulations such as finite element analysis or mechanistic approaches. Trabecular bone of the femoral head of healthy and osteoporotic patients can thus be modeled independently from age or osteoporosis, but as an elasto‐visco‐plastic material. Because only trabeculae from the femoral head were investigated in tensile mode here, further research is necessary that focusses on different anatomical locations (e.g., spine and radius) and different loading states (e.g., compression and bending) to verify these observations for cancellous bone in a more general manner.

## Author Contributions

**Martin Frank:** Formal analysis; investigation; methodology; validation; visualization; writing‐original draft. **Andreas Reisinger:** Formal analysis; software; writing‐review & editing. **Dieter Pahr:** Supervision; writing‐review & editing. **Phillipp Thurner:** Conceptualization; resources; supervision; writing‐review & editing.

## Authorsʼ roles

Study design: PJT, DHP, and MF. Study conduct: MF and AGR. Data collection: PJT and MF. Data analysis: MF and AGR. Data interpretation: MF, AGR, DHP, and PJT. Drafting manuscript: MF. Revising manuscript content: AGR, DHP, and PJT. Approving final version of manuscript: MF, AGR, DHP, and PJT. MF takes responsibility for the integrity of the data analysis.

## Conflict of Interest

All authors filled out the ICMJE Form for Disclosure of Potential Conflicts of Interest. Dieter H. Pahr discloses involvement in Dr. Pahrs Ingenieurs e.U. All other authors state that they have no conflicts of interest.

## Data Accessibility Statement

Additional data (raw data of all stress–strain curves, evaluation of apparent mechanical properties and evaluation of material properties with the rheological model) can be found at: https://data.mendeley.com/datasets/b8yfxmjfrz/draft?a=3a59f6f7-7738-4caa-88f8-7533f07c2621 Frank, Martin (2020), “Trab_Osteo_Stress‐strain”, Mendeley Data, V1, doi: 10.17632/b8yfxmjfrz.1

### Peer Review

The peer review history for this article is available at https://publons.com/publon/10.1002/jbm4.10503.

## Supporting information


**Supplementary Information S1.** Supplementary Information.
