# Supplementary material for: Effects of Osteoporosis on Bone Morphometry and Material Properties of Individual Human Trabeculae in the Femoral Head
Source: JBMR Plus. 2021 May 4;5(6):e10503. doi: 10.1002/jbm4.10503 (PMC8216141; doi:10.1002/jbm4.10503)
Supplement: Supplementary file 1 — Supplementary Information S1. Supplementary Information. [file JBM4-5-e10503-s001.pdf]

# Supplemental information

## Study population

Age, sex, femur side, BMI, *T* score, and FRAX score (without aBMD) for each donor are listed in Table S 1. Both groups (osteoporotic and control) contained five female and five male donors, respectively.

Table S 1: Study population. FRAX: fracture risk assessment tool

| Donor | Age/y | sex | side | BMI/(kgm$^{-2}$) | *T* score | FRAX score/% |
|---|---|---|---|---|---|---|
| O24 | 66 | F | L | 27.1 | -2.6 | 4.0 |
| O25 | 60 | M | L | 29.1 | -0.4 | 0.5 |
| O30 | 77 | F | R | 34.3 | -2.2 | 28 |
| O33 | 79 | M | R | 30.8 | -2.3 | 2.2 |
| O45 | 84 | F | L | 29.0 | -2.1 | 25.0 |
| O48 | 90 | M | R | 23.7 | -2.3 | 10.0 |
| O42 | 88 | M | L | | -3.2 | 29.0 |
| O39 | 70 | F | L | 21.2 | -2.8 | 22.0 |
| O41 | 73 | M | R | 20.4 | -2.8 | 15.0 |
| O35 | 59 | F | L | 19.5 | -3.4 | 3.3 |
| **Osteoporotic mean** | **74.6** | | | **26.1** | **-2.4** | **13.9** |
| A2439 | 61 | F | L | 36.6 | -0.5 | 0.7 |
| A2440 | 63 | M | L | 30.8 | 6.0 | 1.0 |
| A2441 | 63 | F | R | 25.5 | 1.9 | 1.5 |
| A2444 | 65 | M | L | 50.3 | 3.3 | 0.5 |
| A4167 | 70 | M | R | 29.0 | -1.8 | 1.0 |
| A4175 | 80 | M | R | 16.7 | -1.9 | 6.3 |
| A4173 | 81 | M | R | 22.4 | -0.2 | 3.6 |
| 56003898 | 83 | F | R | 31.0 | 3.1 | 11.0 |
| A2446 | 72 | F | L | 24.7 | -2.7 | 4.5 |
| A4171 | 57 | F | R | 33.6 | 4.0 | 0.6 |
| **Control mean** | **69.5** | | | **30.1** | **1.1** | **3.1** |

## Clinical baseline characteristics – boxplots

Figure S 1 illustrates boxplots of the *T* score and the FRAX score (exclusive aBMD), reported in the manuscript in Table 1. Both parameters are significantly changed with osteoporosis, as well in the fracture-based classification, as in the *T* score based one (FRAX close to significance, p = 0.051).

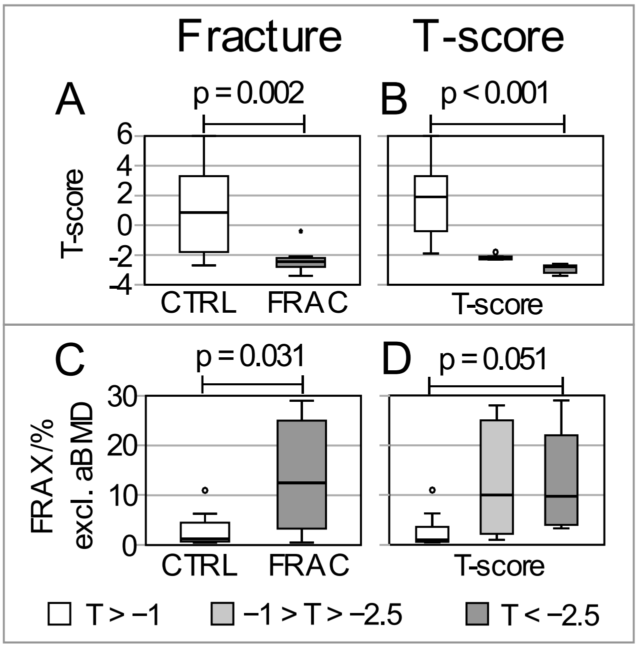


Figure S 1: Clinical data is shown for grouping based on the fracture criterion (left, panels *A* & *C*) and on the *T* score (right, panels *B* & *D*). (*A*, *B*) *T* score is significantly lower in the fracture group and in $T < -2.5$. (*C*, *D*) FRAX (excl. aBMD) is significantly larger in the fracture group and almost significant in group $T < -2.5$ ($p = 0.051$). o Mild outlier (< 3.0×IQR), * Extreme outlier (≥ 3.0×IQR).

## Material and mechanical properties – boxplots & tables

Figure S 2 and Figure S 3 illustrate boxplots of the material properties reported in the main manuscript in Table 2 – top, Figure S 4 the apparent mechanical properties shown in Table 2 – bottom. These boxplots are intended to provide a more thorough comparison of the data distributions of obtained properties. Neither in fracture-based classification (see Figure S 2), nor in *T* score based one (see Figure S 3) any significant difference could be determined. In contrast, there was a significant increased yield strain and elastic work in $T < -2.5$, compared to $-1.0 < T < -2.5$ (see Figure S 4). Hereby, elastic work and yield strain behave very similar, as these two variables are highly correlated.

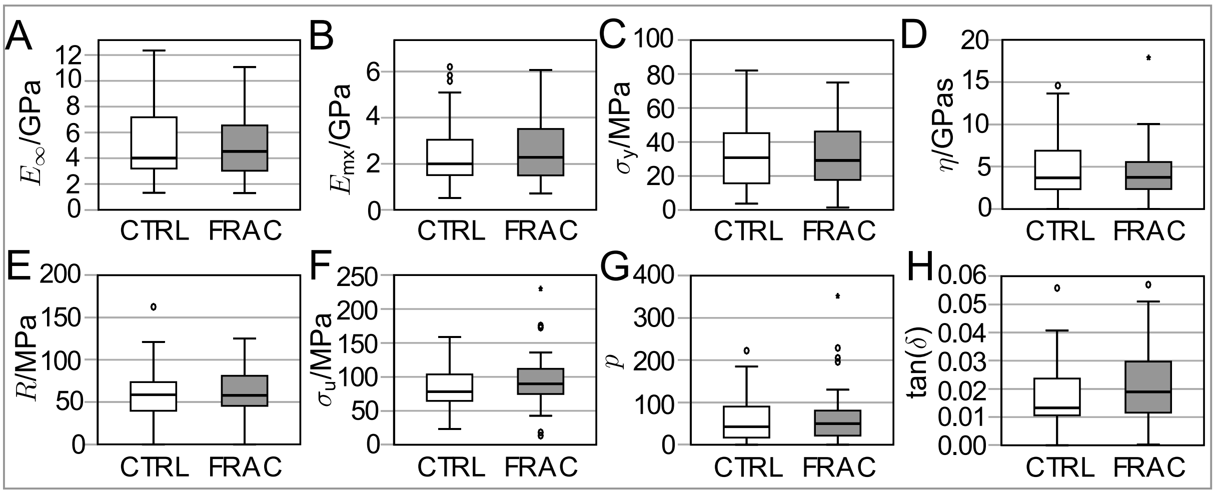


Figure S 2: Material properties determined with the rheological model. Grouping is based on the fracture criterion. (*A*) long term modulus ($E_{\infty}$). (*B*) Maxwell modulus ($E_{mx}$). (*C*) yield stress ($\sigma_y$). (*D*) viscosity ($\eta$). (*E*) hardening stress ($R$). (*F*) ultimate stress ($\sigma_u$). (*G*) hardening exponent ($p$). (*H*) loss tangent ($\tan(\delta)$). o Mild outlier (<3.0×IQR), * Extreme outlier (≥3.0×IQR).

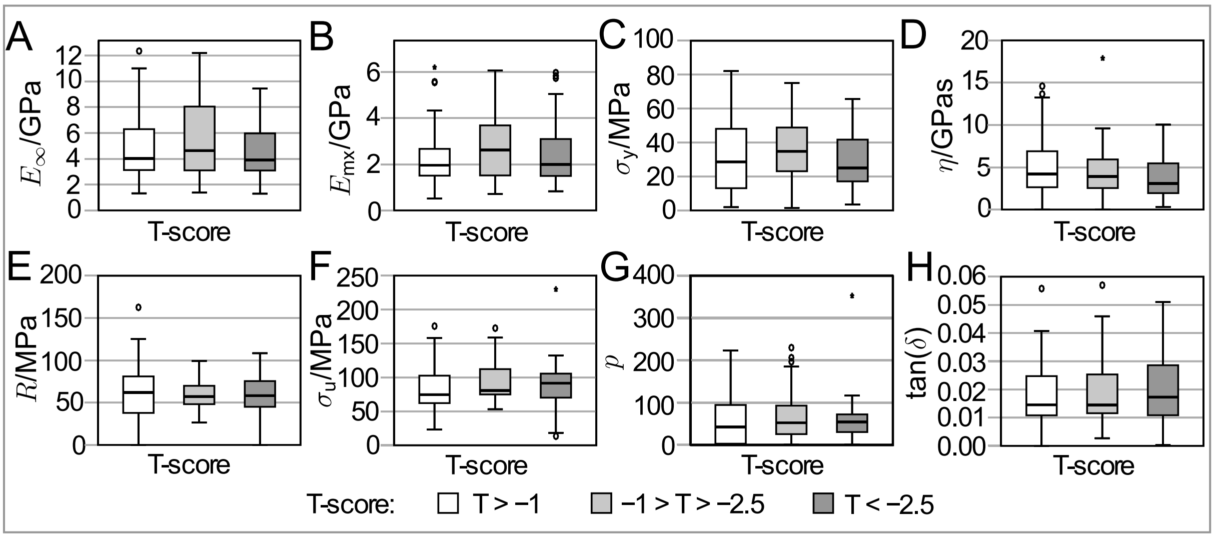


Figure S 3: Material properties determined with the rheological model. Grouping is based on the *T* score. (*A*) long term modulus ($E_{\infty}$). (*B*) Maxwell modulus ($E_{mx}$). (*C*) yield stress ($\sigma_y$). (*D*) viscosity ($\eta$). (*E*) hardening stress ($R$). (*F*) ultimate stress ($\sigma_u$). (*G*) hardening exponent ($p$). (*H*) loss tangent ($\tan(\delta)$). o Mild outlier (<3.0×IQR), * Extreme outlier (≥3.0×IQR).

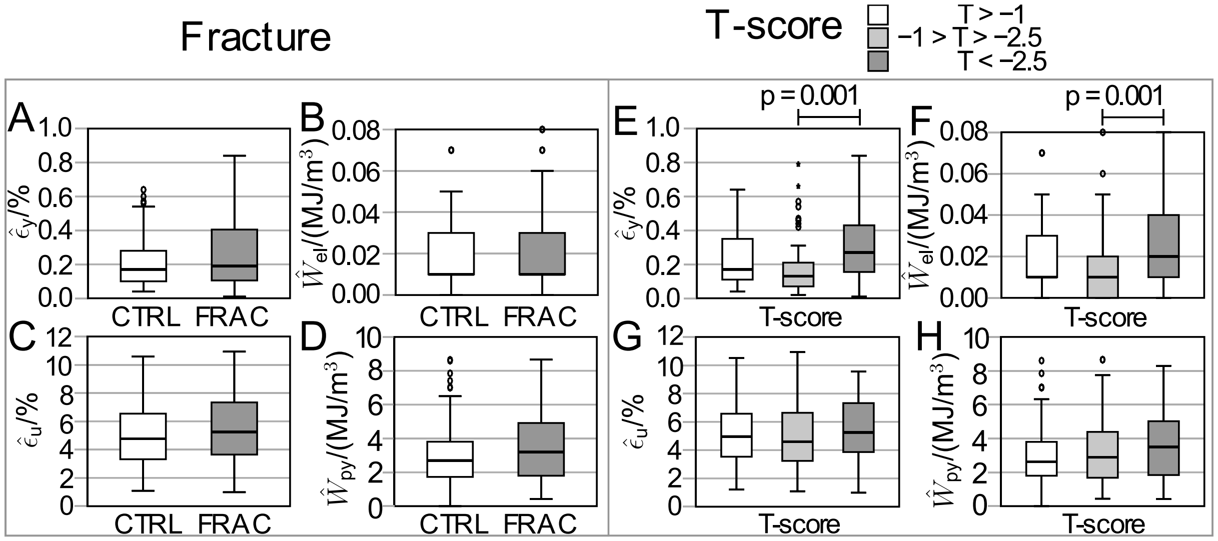


Figure S 4: Apparent mechanical properties determined with curve-fitting on the envelope curve. Grouping is based on the fracture criterion (left, panels *A* – *D*) and on the *T* score (right, panels *E* – *H*). (*A*, *E*) yield strain ($\varepsilon_y$). (*B*, *F*) Elastic work ($W_{el}$). (*C*, *G*) ultimate strain ($\varepsilon_u$). (*D*, *H*) post yield work ($W_{py}$). o Mild outlier (<3.0×IQR), * Extreme outlier (≥3.0×IQR).

Table S 2 shows the tensile (tangent) stiffnesses of the first three loading and unloading cycles. Here, unloading stiffness was significantly larger than loading stiffness in the first three cycles, but there was no significant difference between the control and fracture group.

Table S 2: Determined tensile (tangent) stiffnesses of the first three loading and unloading cycles of the control and fracture group. $E_{load}$: Tensile stiffness in loading cycle, $Eun_{load}$: Tensile stiffness in unloading cycle.

| | $E_{load}$/GPa | | $E_{unload}$/GPa | | p value CTRL | p value FRAC |
|---|---|---|---|---|---|---|
| **Cylce** | **CTRL** | **FRAC** | **CTRL** | **FRAC** | | |
| 1 | 7.4 ± 4.2 | 7.9 ± 4.7 | 21.4 ± 12.3 | 18.8 ± 13.4 | **< 0.001** | **< 0.001** |
| 2 | 8.4 ± 4.6 | 8.9 ± 5.6 | 15.1 ± 8.5 | 13.3 ± 8.2 | **< 0.001** | **0.001** |
| 3 | 9.8 ± 5.2 | 9.5 ± 5.2 | 10.7 ± 5.2 | 10.9 ± 5.4 | 0.282 | |

Table S 3 illustrates the material properties determined with the rheological model and the mechanical properties determined with curve fitting on the envelope curve based on trabecular orientation. No significant difference was observed in any property, except a significantly ($p < 0.05$) smaller apparent yield strain and elastic work in longitudinal oriented trabeculae.

Table S 3: Material properties determined with the rheological model (top) and mechanical properties determined with curve fitting on the envelope curve (bottom) based on trabecular orientation. $E_\infty$: long-term modulus, $E_{mx}$: Maxwell elastic modulus, $\sigma_y$: yield stress, $p$: exponential hardening coefficient, $R$: hardening stress ($R = \sigma_u - \sigma_y$), $\sigma_u$: ultimate stress, $\eta$: viscosity, tan $\delta$: loss tangent, $\hat{E}$: apparent stiffness, $\hat{\sigma}_y$: apparent yield strain, $\hat{\sigma}_u$: apparent ultimate strain, $\widehat{W}_{py}$: apparent post-yield work, $\widehat{W}_{el}$: apparent elastic work. Mean values are indicated ± standard deviation. Significant p-values ($p < 0.05$) are highlighted bold.

| | **Orientation** | | |
|---|---|---|---|
| **Parameter** | **transversal** | **longitudinal** | **p** |
| $E_\infty$/GPa | 4.9 ± 2.5 | 5.0 ± 2.7 | 0.929 |
| $E_{mx}$/GPa | 2.5 ± 1.6 | 2.5 ± 1.2 | 0.750 |
| $\sigma_y$/MPa | 31.6 ± 19.3 | 31.2 ± 18.7 | 0.942 |
| $p$ | 66.8 ± 66.9 | 59.6 ± 58.4 | 0.572 |
| $R$/MPa | 57.7 ± 30.6 | 62.3 ± 26.5 | 0.717 |
| $\sigma_u$/MPa | 85.8 ± 38.9 | 91.8 ± 30.5 | 0.335 |
| $\eta$/GPas | 4.7 ± 3.4 | 4.4 ± 3.5 | 0.717 |
| tan $\delta$ | 0.018 ± 0.011 | 0.020 ± 0.013 | 0.672 |
| $\hat{E}$/GPa | 8.0 ± 5.0 | 8.2 ± 4.7 | 0.648 |
| $\hat{\varepsilon}_y$/% | 0.28 ± 0.20 | 0.21 ± 0.17 | **0.012** |
| $\hat{\varepsilon}_u$/% | 5.1 ± 2.1 | 5.3 ± 2.5 | 0.958 |
| $\widehat{W}_{py}$/(MJ/m$^3$) | 3.2 ± 1.9 | 3.3 ± 2.0 | 0.865 |
| $\widehat{W}_{el}$/(MJ/m$^3$) | 0.024 ± 0.022 | 0.016 ± 0.017 | **0.003** |

# Tissue mineral density (TMD) – boxplots & correlations

Figure S 5 – A & B illustrate the mean intensity profile of all samples for *T* score based classification (A) and trabecular orientation (B) (mean solid, 95% confidence interval shaded) across the mass centroid axis (normalized), as illustrated in the main manuscript in insets in Figures 4 A & B. Figure S 5 – C & D demonstrate the normalized histogram of TMD distribution of all samples for *T* score based classification (C) and trabecular orientation (D) (mean solid, 95% confidence interval shaded) for whole individual trabeculae. Here, donors with osteopenia showed a significantly larger TMD than control and osteoporotic donors. Similarly, longitudinal trabeculae exhibited a significantly larger TMD than transversal samples.

Figure S 6 demonstrates that in fracture-based classification fracture and control trabeculae exhibited a significant negative correlation of mean TMD with apparent ultimate strain.

However, this behavior was more dominant in control samples, in comparison to osteoporotic ones.

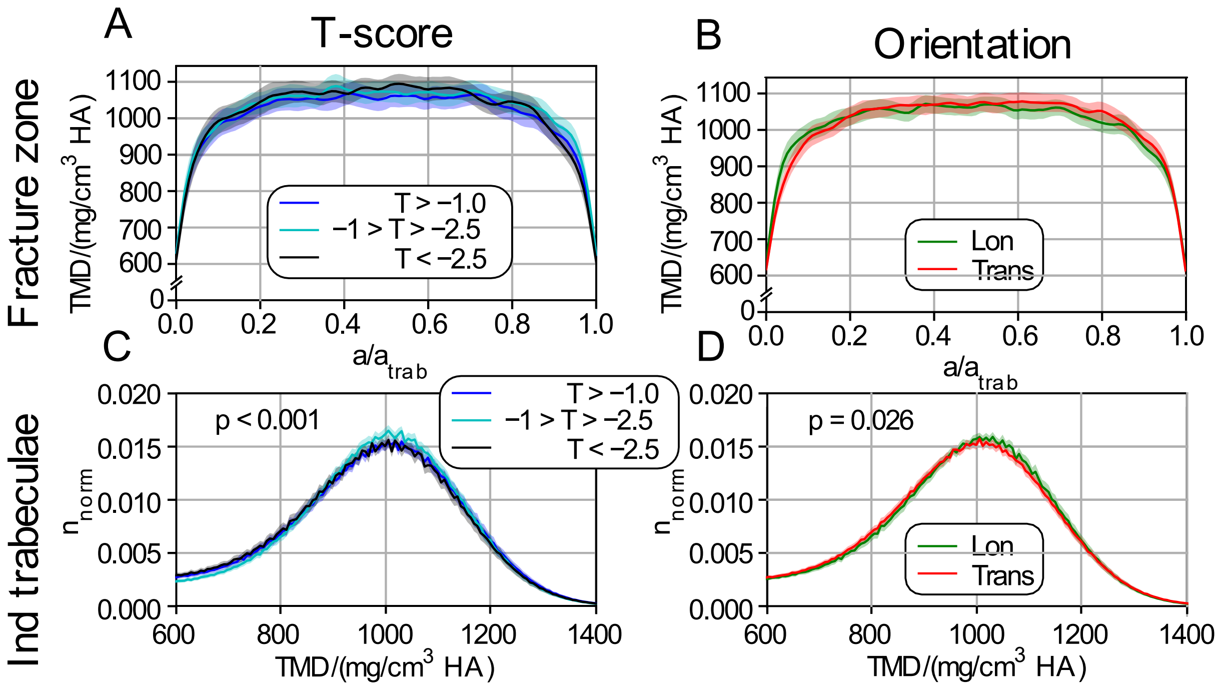


Figure S 5: (*A* & *B*): Mean intensity profile of all samples for *T* score based classification (*A*) and trabecular orientation (*B*) (mean solid, 95% confidence interval shaded) across the mass centroid axis (normalized), as illustrated in insets in Figures 4 *A* & *B*. (*C* & *D*):Normalized histogram of TMD distribution of all samples for *T* score based classification (*C*) and trabecular orientation (*D*) (mean solid, 95% confidence interval shaded) for whole individual trabeculae (each value corresponds to one voxel obtained with µCT).

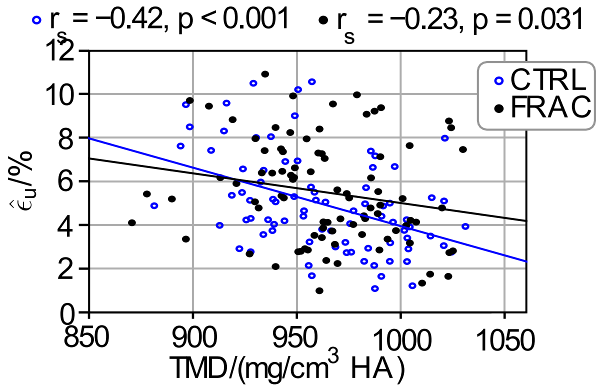


Figure S 6: Correlation plot of mean TMD with apparent post-yield work ($\hat{\varepsilon}_u$). Correlations are shown for fracture-based classification (CTRL blue, FRAC black), with actual spearman rank correlation coefficients and corresponding p values.

# Bone morphometry – boxplots & representative bone sphere slices

Figure S 7 and Figure S 8 show boxplots of the bone morphometry parameters reported in the main manuscript in Table 4. As with mechanical data, boxplots are provided for a better representation of data distribution. Bone surface (BS) and trabecular number (Tb.N) were significantly smaller in osteoporotic fractured patients (see Figure S 7). Similarly, bone surface and trabecular number were significantly smaller, but trabecular separation (Tb.Sp) was significantly larger, in patients with lower *T* scores (*T* < -1.0, see Figure S 8). Further, bone volume to total volume (BVTV) was significantly smaller in *T* < -2.5, whereas the decrease in the fracture classification was not significant (p = 0.186). Figure S 9 demonstrates representative cross-sections of bone spheres used for bone morphometry. Here, it can be observed that there is a decrease in bone volume to total volume and trabecular number, accompanied by an increase in trabecular separation with increasing *T* scores.

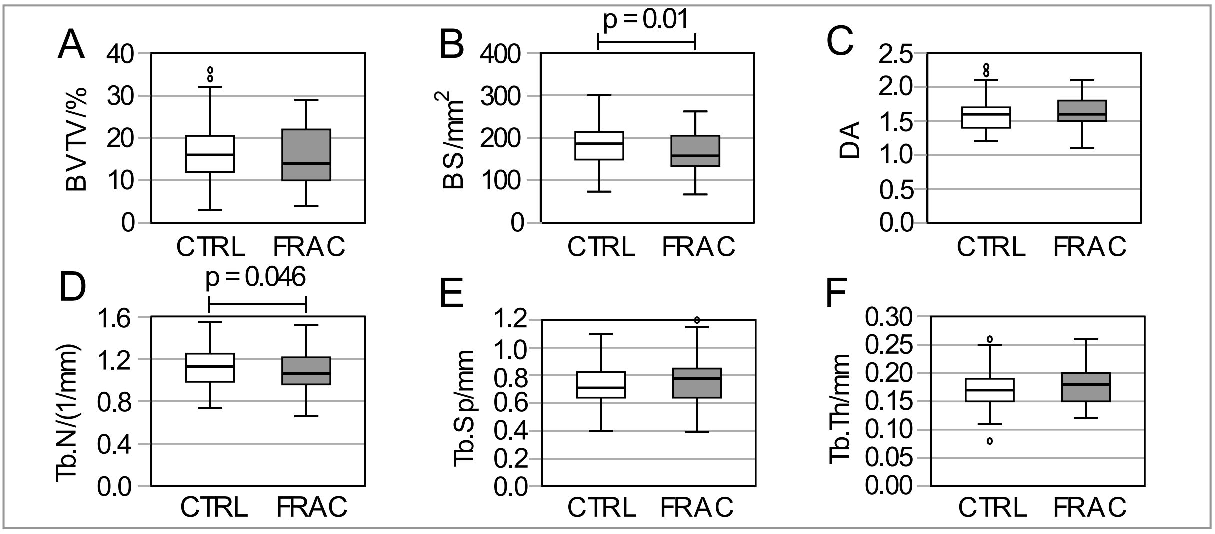


Figure S 7: µCT-derived bone morphometry. Grouping is based on the fracture criterion. (*A*) Bone volume to total volume (BVTV). (*B*) Bone surface (BS). (*C*) Degree of anisotropy (DA). (*D*) Trabecular number (Tb.N). (*E*) Trabecular separation (Tb.Sp). (*F*) Trabecular thickness (Tb.Th). o Mild outlier (<3.0×IQR), * Extreme outlier (≥3.0×IQR).

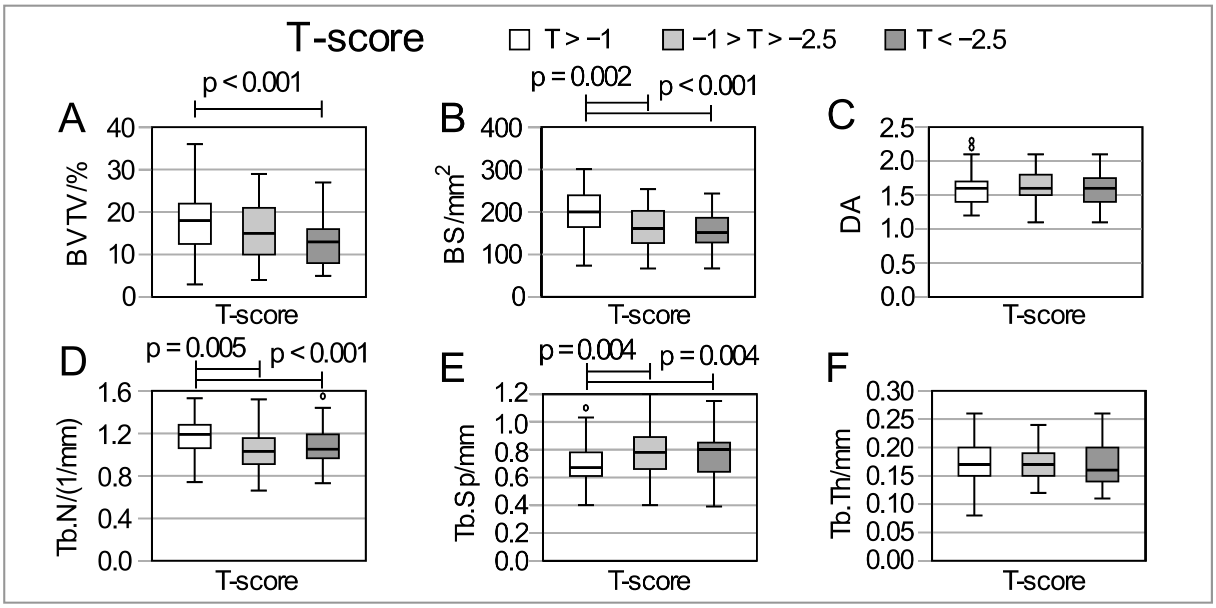


Figure S 8: µCT-derived bone morphometry. Grouping is based on the *T* score. (*A*) Bone volume to total volume (BVTV). (*B*) Bone surface (BS). (*C*) Degree of anisotropy (DA). (*D*) Trabecular number (Tb.N). (*E*) Trabecular separation (Tb.Sp). (*F*) Trabecular thickness (Tb.Th). o Mild outlier (<3.0×IQR), * Extreme outlier (≥3.0×IQR).

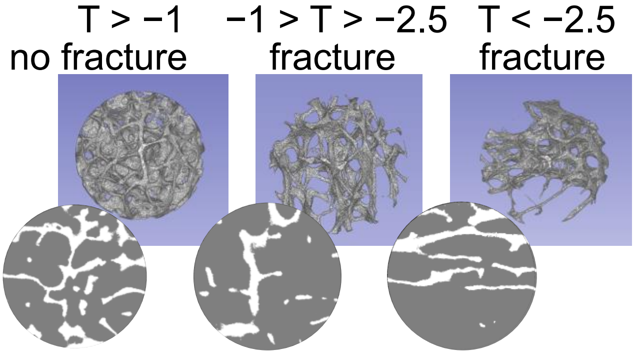


Figure S 9: Representative cross-sectional slices of bone spheres, used for µCT-derived bone morphometry. All spheres were oriented according to their three largest eigenvectors, to show the same orientation (all shown spheres were dissected from the longitudinal/ trajectory group). (Left) No fracture, *T* > -1. (Middle) fracture and -1 > *T* > -2.5. (Right) fracture and *T* < -2.5.
